# Application Value of Magnetic Resonance Radiomics and Clinical Nomograms in Evaluating the Sensitivity of Neoadjuvant Chemotherapy for Nasopharyngeal Carcinoma

**DOI:** 10.3389/fonc.2021.740776

**Published:** 2021-11-01

**Authors:** Chunmiao Hu, Dechun Zheng, Xisheng Cao, Peipei Pang, Yanhong Fang, Tao Lu, Yunbin Chen

**Affiliations:** ^1^ Department of Radiology, Fujian Medical University Cancer Hospital, Fujian Cancer Hospital, Fuzhou, China; ^2^ Department of Pharmaceuticals Diagnosis, GE Healthcare, Hangzhou, China

**Keywords:** nasopharyngeal carcinoma, radiomics, magnetic resonance imaging, neoadjuvant chemotherapy, efficacy evaluation

## Abstract

**Objective:**

To predict the sensitivity of nasopharyngeal carcinoma (NPC) to neoadjuvant chemotherapy (NACT) based on magnetic resonance (MR) radiomics and clinical nomograms prior to NACT.

**Materials and Methods:**

From January 2014 to July 2015, 284 consecutive patients with pathologically confirmed NPC underwent 3.0 T MR imaging (MRI) before initiating NACT. The patients’ data were randomly assigned to a training set (n = 200) or a test set (n = 84) at a ratio of 7:3. The clinical data included sex, tumor (T) stage, lymph node (N) stage, American Joint Committee on Cancer (AJCC) stage, and the plasma concentration of Epstein–Barr virus (EBV) DNA. The regions of interest (ROI) were manually segmented on the axial T2-weighted imaging (T2WI) and enhanced T1-weighted imaging (T1WI) sequences using ITK-SNAP software. The radiomics data were post-processed using AK software. Moreover, the Maximum Relevance Minimum Redundancy (mRMR) algorithm and the Least Absolute Shrinkage and Selection Operator (LASSO) were adopted for dimensionality reduction to screen for the features that best predicted the treatment efficacy, and clinical risk factors were used in combination with radiomics scores (Rad-scores) to construct the clinical radiomics-based nomogram. DeLong’s test was utilized to compare the area under the curve (AUC) values of the clinical radiomics-based nomogram, radiomics model, and clinical nomogram. Decision curve analysis (DCA) was employed to evaluate each model’s net benefit.

**Results:**

The clinical nomogram was constructed based on data from patients who were randomly assigned according to T2WI and enhanced T1WI sequences. In the training set, the T2WI sequence-based clinical radiomics nomogram and the radiomics model outperformed the clinical nomogram in predicting the NACT efficacy (AUC, 0.81 *vs*. 0.60, *p* = 0.001279 and 0.76 *vs*. 0.60, *p* = 0.03026). These findings were well-verified in the test set. The enhanced T1WI sequence-based clinical radiomics nomogram exhibited better performance in predicting treatment efficacy than the clinical nomogram (AUC, 0.79 *vs*. 0.62, respectively; *p* = 0.0000834). The DCA revealed that the T2WI and clinical radiomics-based nomograms resulted in a net benefit in predicting the NACT efficacy.

**Conclusion:**

The clinical radiomics-based nomogram improved the prediction of NACT efficacy, with the T2WI sequence-based clinical radiomics achieving the best effect.

## Introduction

Radiotherapy is the main treatment for nasopharyngeal carcinoma (NPC), which is associated with a complicated anatomical structure. About 70% of patients are at the local stages (stages III–Va) (1), and about 10%–15% of patients with locally advanced NPC will develop a primary lesion or regional lymph node residue, or experience disease recurrence (2, 3). To reduce the likelihood of local recurrence and distant metastasis (DM) of NPC, chemotherapy in combination with radiotherapy is the main recommendation at present (4). The chemotherapy regimens include neoadjuvant, concurrent, and adjuvant chemotherapy; among these options, neoadjuvant chemotherapy (NACT) has been increasingly recommended due to its tolerability and its ability to induce the early elimination of micrometastases (5–8). The administration of NACT prior to concurrent chemotherapy can improve the disease-free survival (DFS) and overall survival (OS) of patients with NPC (9).

The early prediction of a patient’s response to NACT is of crucial importance, as it assists clinicians in formulating suitable therapeutic regimens before treatment and minimizes unnecessary chemotherapy-induced toxicity. At present, the magnetic resonance (MR) technologies used to predict the therapeutic effect of NACT include conventional MR imaging (MRI), diffusion-weighted imaging (DWI), dynamic contrast-enhanced MR (DCE-MR) imaging, and intravoxel incoherent motion (IVIM) and diffusion kurtosis imaging (DKI) (10–12). When evaluating the therapeutic effect of a treatment for NPC, the aforementioned functional MR technologies assess the tumor mass from the level of interest, rather than from the overall mass, and there are differences in the parameters measured by different equipment. Recent studies have shown that NPC survival is related to the clinical tumor, node, metastasis (TNM) stage, the plasma levels of Epstein-Barr virus (EBV) DNA, the EBV antibody levels evaluated by assays, the lactic dehydrogenase (LDH) and hemoglobin (HGB) levels, the platelet count (PLT), and the lymphocyte (LYM) and neutrophil (NEUT) ratios. NPC survival is also closely related to a patient’s sensitivity to chemotherapeutic agents and radiotherapy (13), while tumor heterogeneity is related to tumor invasion and resistance (14).

At present, there are few effective tools to accurately predict a patient’s response to NACT. However, radiomics can describe the structural features and heterogeneity of tissue, which are closely related to histopathological, proteomic, and genetic heterogeneity, and are associated with tumor cell structure, angiogenesis, and necrosis (15–18). Recent studies have shown that employing a combination of MR-based radiomics with multiple machine learning techniques can help predict the diagnosis of NPC and evaluate the therapeutic effects of treatment (19, 20).

Based on features related to tumor heterogeneity and the efficacy of NACT identified from the MR-based multisequencing radiomics conducted prior to the initiation of NACT, this study aimed to construct a nomogram based on NPC clinic data, EBV-DNA levels, and important laboratory indexes as a function of radiomics to predict the sensitivity of NPC to NACT.

## Materials and Methods

### Clinical Case Data

From January 2014 to July 2015, 284 consecutive patients with pathologically confirmed NPC were selected for this study, which was approved by the institutional ethics committee (YKT2021-012-01). Each patient provided written informed consent before examination. Data from 220 male and 64 female patients were randomly assigned to either the training set (n = 200) or the test set (n = 84) at a ratio of 7:3. The training set comprised data from 155 male and 45 female patients, with an average age of 47.1 ± 11.3 years. The test set comprised data from 65 male and 19 female patients, with an average age of 47 ± 11.0 years. According to the World Health Organization (WHO) classification system (21), NPC cases were pathologically classified as type I (differentiated, chancroid), type II (differentiated, non-chancroid), and type III (undifferentiated, non-chancroid). There were four type I, eight type II, and 262 type III cases included in the present study.

All enrolled patients underwent MR examinations before treatment and at the end of the second cycle of NACT. For 5 years after the completion of treatment, all patients received systemic follow-up examinations. The patient inclusion criteria were as follows: (a) patients with locally advanced (stages III–IVa), pathologically confirmed NPC; (b) patients who were treatment naive; (c) patients who underwent re-examination by MR techniques following two cycles of NACT; (d) patients who underwent nasopharyngeal MR examination before and after receiving comprehensive treatment; (e) patients with complete follow-up imaging data ([including skull base and neck MRI, lung computed tomography (CT), abdominal B-scan ultrasonography, bone emission computed tomography (ECT) or systemic positron emission tomography (PET)/CT) that demonstrated no recurrence within 5 years of treatment; and (f) patients with pathologically confirmed local recurrence, DM, or obvious metastasis revealed on multiple imaging examinations. Based on the latter criterion, patients with local recurrence and DM were classified in the recurrence group, whereas those without local recurrence or DM within 5 years of treatment were classified in the non-recurrence group. The exclusion criteria were as follows: (a) patients with incomplete image sequences or with images whose quality was insufficient for assessing the diagnostic criteria and (b) patients who were lost to follow-up.

### Therapeutic Regimen

The same therapeutic regimen—namely, chemotherapy plus intensity-modulated radiation therapy (IMRT)—was adopted for all the enrolled patients. More specifically, all patients received two cycles of NACT, followed by IMRT. Additionally, all the enrolled patients received systemic examinations, including nasopharyngoscopy, serology (EBV-DNA quantification, EBV antibody assays, and routine blood and biochemical analyses), enhanced MRI of the nasopharynx/skull base/neck, multilayer helical enhanced CT of the chest, whole abdominal ultrasonography, and systemic bone ECT; in addition, some patients underwent systemic PET/CT examinations. TNM classification was conducted in accordance with the Union for International Cancer Control (UICC)/American Joint Committee on Cancer (AJCC) classification system (the 8th edition) (4).

The NACT consisted of two cycles of 21 days each. A platinum-based regimen in combination with gemcitabine or paclitaxel was adopted. The specific regimen was as follows: 80–100 mg/m^2^ nedaplatin (Qilu Pharmaceutical Co., Ltd., Shandong, China, on day 2) + 135 mg/m^2^ paclitaxel (Hainan Sinochem United Pharmaceutical Co., Ltd., Hainan, China, on day 1); 80–100 mg/m^2^ cis-platinum (Qilu Pharmaceutical Co., Ltd., Shandong, China, on days 1–3) + 135 mg/m^2^ paclitaxel (on day 1), 80–100 mg/m^2^ nedaplatin (on day 1) or 80–100 mg/m^2^ cis-platinum (on days 1–3) + 1,000 mg/m^2^ gemcitabine (Jiangsu Haosoh Pharmaceutical Co., Ltd., on days 1 and 8).

### NPC Target Volume Delineation

The IMRT target volumes (22) were delineated. The NPC gross tumor volume (GTV) included the primary NPC lesion and the affected tissues, the retropharyngeal lymph node, and the neck lymph node metastatic region. Clinical target volume 1 (CTV1) was defined as the external expansion of GTV1 by 5–10 mm + the corresponding nasopharyngeal mucosa and submucosa by 5 mm. CTV2 included CTV, which appropriately considered the adjacent structures affected by the extent and location of tumor invasion, and the structures surrounding the nasopharynx and skull base. CTVnd included the metastatic lymph nodes in the neck and the lymph node drainage region. The planning target volume (PTV) included the above-mentioned target volumes expanded externally by 3 mm. The recommended prescription doses were as follows: the total doses of PGTV_NX_ and PGTV_RPN_ were 66–76 Gy; the total dose of PGTVnd was 66–70 Gy; the total dose of PCTV1 was 60–62 Gy; and the total doses of PCTV2 and PCTVnd were 50–56 Gy.

### Evaluation of the Therapeutic Effect of NACT

Lesion regression after NACT was evaluated according to the Response Evaluation Criteria in Solid Tumors (RECIST) and rated as complete response (CR), partial response (PR), stable disease (SD), and progressive disease (PD) (23). Tumor regression was evaluated in each patient after the second NACT cycle; those who received a PR or CR classification were categorized as the response group, whereas those with an SD or PD classification were categorized as the non-response group.

### Imaging Method and Model Construction

An Achieva 3.0 T TX MR scanner (Philips Medical Systems, The Netherlands) was adopted for scanning, with a 16-channel head–neck combined coil.

For the turbo spin echo (TSE) T2-weighted imaging (T2WI) sequence, the short inversion time inversion recovery (STIR) sequence was adopted for fat suppression. The following setting parameters were used: field of view (FOV) = 230 × 260 mm, layer thickness = 5 mm, interlayer spacing = 1 mm, repetition time (TR) = 7,620 ms, echo time (TE) = 60 ms, matrix = 290 × 174, number of excitations (NEX) = 2, inversion time (TI) = 230 ms, echo train length (ETL) = 17, and number of scanning slices = 37.

For the transverse enhanced T1WI sequence, the following setting parameters were used: FOV = 230 × 260 mm, layer thickness = 5 mm, interlayer spacing = 1 mm, TR = 500 ms, TE = 8 ms, matrix = 232 × 217, NEX = 2, ETL = 5, and number of scanning slices = 37. Gadopentetate dimeglumine (Gd-DTPA, 0.1–0.2 mmol/kg) was injected *via* the elbow vein with a high-pressure injector.

The upper bound of the NPC scanning region was the inferior temporal lobe, whereas the lower bound was the superior thoracic aperture.

### Data Collection

All the transverse T2WI sequences and enhanced T1WI sequences were collected before patients initiated NACT. The original images were retrieved from the Picture Archiving and Communication System (PACS, Shida Technological Medical System) and saved in the Digital Imaging and Communications in Medicine (DICOM) format. The regions of interest (ROIs) in the above-mentioned image sequences were manually segmented using the open source ITK-SNAP software (v.3.4.0, http://www.itksnap.org). The ROIs represented the tumor profiles for each transverse view. The lesion profiles were independently delineated by one radiologist with 10 years of experience and one radiologist with 20 years of experience to obtain the 3D volume of the ROI of each lesion. Thereafter, the intraclass correlation coefficient (ICC) for the measurer and the between-measurements ICC were calculated. Later, 50 cases were selected randomly, and the respective ROIs were delineated by the radiologist with 10 years of experience; 1 week later, the ROIs were delineated again to evaluate the ICC of the same measurer. In addition, the ROIs were delineated by the radiologist with 20 years of experience to evaluate the intergroup ICC. An ICC > 0.75 was considered to be indicative of a favorable degree of consistency.

### Histological Feature Extraction

AK software (Artificial Intelligence Kit V3.0.0.R, GE Healthcare) was employed for post-processing of the radiomic data. More specifically, the ROIs were delineated using the open source ITK-SNAP software and subsequently imported into the AK software. The first-order features, morphological features, gray-level co-occurrence matrix (GLCM), gray-level run length matrix (GLRLM), gray-level size zone matrix (GLSZM), and wavelet features were chosen to extract a total of 680 features.

### Feature Selection

Two feature selection methods were used, including the Maximum Relevance Minimum Redundancy (mRMR) algorithm and the Least Absolute Shrinkage and Selection Operator (LASSO). First, mRMR was executed to eliminate the redundant and irrelevant features, and 20 features were retained. Subsequently, LASSO analysis was conducted, and 10-fold cross-validation was utilized to select the optimal features and the λ parameter to obtain the compression model coefficient. The optimized feature subset was selected to construct the final model. In total, 19 features were selected from the transverse T2WI sequence and 19 from the transverse enhanced T1WI sequence.

### Radiomics Model Construction

**Figure 1:**
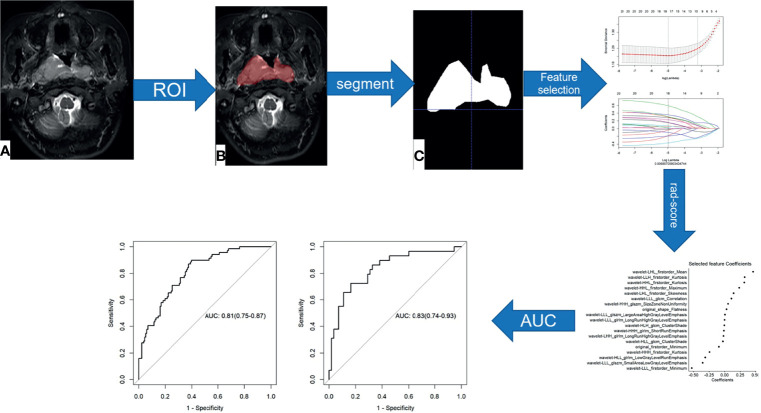
Flow chart of radiomics feature selection and model construction. **(A–C)** Image segmentation is performed on MRI images.

The radiomics scores (Rad-scores) of the training cohort were weighted according to the combination formula constructed from the selected features and the LASSO coefficients in the respective feature training cohorts; in other words, the Rad-score was weighted based on the selected radiomics features and the respective coefficients. The accuracy of the Rad-score (24) in predicting the sensitivity of NPC to NACT was evaluated using the area under curve (AUC) of the receiver operator characteristic (ROC) curve. The methods for the radiomics feature selection and model construction are presented in **Figure 1**.

### Clinical Nomogram Construction and Validation

The clinical risk factors, including age, sex, pathological stage, T (tumor) stage, N (node) stage, AJCC stage, the plasma EBV-DNA, EBV antibody, LDH, and HGB levels, and the PLT count were used in combination with the Rad-score to construct the clinical nomogram. The DeLong test was utilized to compare the differences in the ROC curves among the clinical radiomics nomogram, the radiomics model, and the clinical model. The model identification efficacy was analyzed using the AUCs, whereas the nomogram performance was evaluated using decision curve analysis (DCA) (25). DCA determines the value of the predictive model based on the theoretical relationship between the disease threshold probability and the relative value of the number of false positives to the number of false negatives. The net benefit is the proportion calculated by subtracting the false positive count from the true positive count; then, the relative risk (RR) ratio is weighted; and, finally, the relative hazard ratio (HR) of the false positive and false negative results is weighted. The formula is as follows (Equation 1):


(1) $$Net\text{ Benefit }=\frac{TruePositiveCount}{n}-\frac{FalsepositiveCout}{n}\left(\frac{p_{t}}{1-p_{t}}\right)$$

where “*TruePositiveCount*” and ‘“*FalsePositiveCount*” indicate the numbers of patients with true and false positive results, respectively, “*n*” represents the total number of patients, and “*p_t_*” is the threshold probability, which indicates the predicted therapeutic effect.

### Statistical Analysis

Patients were divided into two groups, including the response group and the non-response group, according to the degree of lesion regression observed after NACT. The Shapiro–Wilk test was adopted to assess whether the data were normally distributed, and measurement data were compared between groups using independent-samples *t*-tests or Mann–Whitney U tests for parametric and non-parametric data, respectively. Enumeration data were analyzed by chi-square tests or Fisher’s exact tests to compare the relationships between two groups. Statistical Package for the Social Sciences (SPSS) 23.0 (SPSS Inc., Chicago, IL,USA) and R software (version 3.5.3; https://www.Rproject.org) were employed for statistical analyses. ICC analyses were conducted to evaluate the internal data reproducibility for the feature extraction of the measurer and between the two measurers. Multivariate logistic regression analysis, ICC, LASSO regression analysis, ROC analysis, the Hosmer–Lemeshow tests, and the DeLong and bootstrap tests were carried out using R software. p < 0.05 indicated that a difference was statistically significant.

## 3 Results

### Clinical Features

There were 284 patients with NPC before the initiation of NACT. After NACT, there were 172 cases in the response group and 112 in the non-response group. The T2WI sequences were randomly divided into a training set (n = 200) and a test set (n = 84). The clinical features of both sets are shown in **Table 1**. There were no significant differences in terms of sex, T stage, AJCC stage, plasma EBV-DNA levels, Rta protein immunoglobulin G antibody (Rta-IgG), viral capsid antigen immunoglobulin A antibody (VCA-IgA), early antigen immunoglobulin A antibody (EA-IgA), and HGB levels or PLT counts between the response group and non-response group in the training set or test set. However, significant differences in age, N stage, and LDH levels between the two groups were observed in the training set.

**Table 1:** Clinical features of patients randomly assigned to the T2-weighted imaging (T2WI) sequence group.

Variable	Level	Training set			Test set		
		0 (n = 121)	1 (n = 79)	*p*	0 (n = 51)	1 (n = 33)	*p*
Sex	Female	30 (24.8%)	15 (19.0%)		14 (27.5%)	5 (15.2%)	
	Male	91 (75.2%)	64 (81.0%)	0.43067	37 (72.5%)	28 (84.8%)	0.2942
Age (years)	Mean (SD)	48.4 (11.6)	45.2 (10.7)	0.04978*	45.8 (11)	48.8 (10.9)	0.2146
T stage	1	0 (0.0)	0 (0.0)		1 (2.0%)	0 (0.0)	
	2	1 (0.8%)	1 (1.3%)		0 (0.0)	0 (0.0)	
	3	67 (55.4%)	31 (39.2%)		22 (43.1%)	7 (21.2%)	
	4	53 (43.8%)	47 (59.5%)	-	28 (54.9%)	26 (78.8%)	-
N stage	0	15 (12.4%)	0 (0.0)		4 (7.8%)	4 (12.1%)	
	1	28 (23.1%)	25 (31.6%)		13 (25.5%)	6 (18.2%)	
	2	66 (54.5%)	45 (57.0%)		28 (54.9%)	16 (48.5%)	
	3	12 (9.9%)	9 (11.4%)	0.01046*	6 (11.8%)	7 (21.2%)	0.5377
AJCC stage	T3	62(51.2%)	29(36.7%)		21 (41.2%)	7 (21.2%)	
	T4a	59(48.8%)	50 (63.3%)	0.084	30 (58.8%)	26 (78.8%)	-
EB-DNA(copy/ml)	Mean (SD)	5449.7 (23055)	3835.6 (10429.2)	0.55915	2122 (5789.9)	8645.3 (36491.9)	0.2090
VCA-IgA(S/CO)	Mean (SD)	3.2 (3.1)	3.2 (2.5)	0.91536	3.1 (2.5)	3.6 (3.2)	0.4078
EA-IgA(S/CO)	Mean (SD)	2.9 (2.9)	2.9 (2.8)	0.93980	2.5 (2.4)	3.1 (3.4)	0.3150
Rta-IgG(S/CO)	Mean (SD)	5.2 (3.5)	5.1 (4.4)	0.82711	5.1 (3.7)	4.4 (3.8)	0.3791
LDH(U/L)	Mean (SD)	155.7 (41.3)	169.2 (52.8)	0.04374*	148.1 (28.7)	158.5 (30.3)	0.1141
HGB(g/L)	Mean (SD)	140.9 (19.3)	144.1 (18.2)	0.23327	145.8 (13.8)	144.1 (15.5)	0.6066
PLT(10E9/L)	Mean (SD)	260.1 (63.8)	257.4 (65.7)	0.76850	245.8 (68)	263.4 (68.5)	0.2482
Rad-score	Median (IQR)	−0.6 (−1.6, 0.1)	0.2 (−0.4, 0.9)	0.0001*	−0.8 (−1.7, 0.1)	0.4 (−0.2, 1.3)	0.0001*

The response group is labeled as 0, whereas the non-response group is labeled as 1. Mean, mean; SD, standard deviation; T, tumor; N, lymph node; AJCC, the American Joint Committee on Cancer; EBV-DNA, plasma Epstein–Barr virus DNA; Rta-IgG, Rta protein immunoglobulin G antibody; VCA-IgA, viral capsid antigen immunoglobulin A antibody; EA-IgA, early antigen immunoglobulin A antibody; HGB, hemoglobin; PLT, platelet; LDH, lactic dehydrogenase; median, median; IQR, interquartile range; Rad-score, radiomics score; -, not available.

*p < 0.05.

The clinical features of the training set and test set for the enhanced T1WI sequence are displayed in **Table 2**. There were no significant differences between the response group and the non-response group in terms of sex, age, T stage, N stage, AJCC stage, plasma EBV-DNA, Rta-IgG, VCA-IgA, EA-IgA, LDH, and HGB levels, or PLT counts in the training set or test set.

**Table 2:** Clinical features of patients randomly assigned to the enhanced T1-weighted imaging (T1WI) sequence group.

Variable	Level	Training set			Test set		
		0 (n = 121)	1 (n = 79)	*p*	0 (n = 51)	1 (n = 33)	*p*
Sex	Female	31 (25.6%)	17 (21.5%)		13 (25.5%)	3 (9.1%)	
	Male	90 (74.4%)	62 (78.5%)	0.62097	38 (74.5%)	30 (90.9%)	0.1129914
Age (years)	Mean (SD)	47.3 (11.3)	45.6 (11.3)	0.29913	48.3 (12)	47.8 (9.6)	0.8348326
T stage	1	1 (0.8%)	0 (0.0)		0 (0.0)	0 (0.0)	
	2	0 (0.0)	1 (1.3%)		1 (2.0%)	0 (0.0)	
	3	64 (52.9%)	29 (36.7%)		25 (49.0%)	9 (27.3%)	
	4	56 (46.3%)	49 (62.0%)	0.06777	25 (49.0%)	24 (72.7%)	-
N stage	0	13 (10.7%)	2 (2.5%)		6 (11.8%)	2 (6.1%)	
	1	28 (23.1%)	19 (24.1%)		13 (25.5%)	12 (36.4%)	
	2	70 (57.9%)	49 (62.0%)		24 (47.1%)	12 (36.4%)	
	3	10 (8.3%)	9 (11.4%)	0.17574	8 (15.7%)	7 (21.2%)	0.5015404
AJCC stage	T3	59 (48.8%)	28 (35.4%)		24 (47.1%)	8 (24.2%)	
	T4a	62 (51.2.1%)	51 (64.6%)	0.11597	27 (52.9%)	25 (75.8%)	-
EB-DNA(copy/ml)	Mean (SD)	4,816.8 (22,454.6)	6,127.5 (25,310.8)	0.70125	3,623.6 (10,297.1)	3,158.4 (6,755.8)	0.8186611
VCA-IgA(S/CO)	Mean (SD)	3.4 (3.2)	3.3 (2.6)	0.80758	2.5 (2.4)	3.4 (3)	0.1425691
EA-IgA(S/CO)	Mean (SD)	2.6 (2.8)	2.9 (3)	0.53971	3 (2.7)	3.1 (2.8)	0.8766902
Rta-IgG(S/CO)	Mean (SD)	5.3 (3.8)	5.2 (4.4)	0.92548	5 (2.9)	4.1 (3.7)	0.2202225
LDH(U/L)	Mean (SD)	153.3 (37.7)	164.9 (47)	0.05398	153.9 (39.3)	168.7 (48.9)	0.1245336
HGB(g/L)	Mean (SD)	142.3 (15)	144 (17.9)	0.47725	142.4 (23.6)	144.5 (16.1)	0.6556982
PLT(10E9/L)	Mean (SD)	256.2 (69.1)	262.3 (63.7)	0.52663	255.2 (55.4)	251.6 (72.7)	0.7978981
Rad-score	Median (IQR)	−0.7 (−1.4, −0.2)	0.2 (−0.3, 0.9)	<0.0001*	−1 (−1.6, −0.4)	0.1 (−0.6, 0.8)	0.0001*

The response group is labeled as 0, whereas the non-response group is labeled as 1. mean, mean; SD, standard deviation; T, tumor; N, lymph node; AJCC, the American Joint Committee on Cancer; EBV-DNA, plasma Epstein–Barr virus DNA; Rta-IgG, Rta protein immunoglobulin G antibody; VCA-IgA, viral capsid antigen immunoglobulin A antibody; EA-IgA, early antigen immunoglobulin A antibody; HGB, hemoglobin; PLT, platelet; LDH, lactic dehydrogenase; median, median; IQR, interquartile range; Rad-score, radiomics score; -, not available.

*p < 0.05.

### Texture Feature Analysis

**Figure 2:**
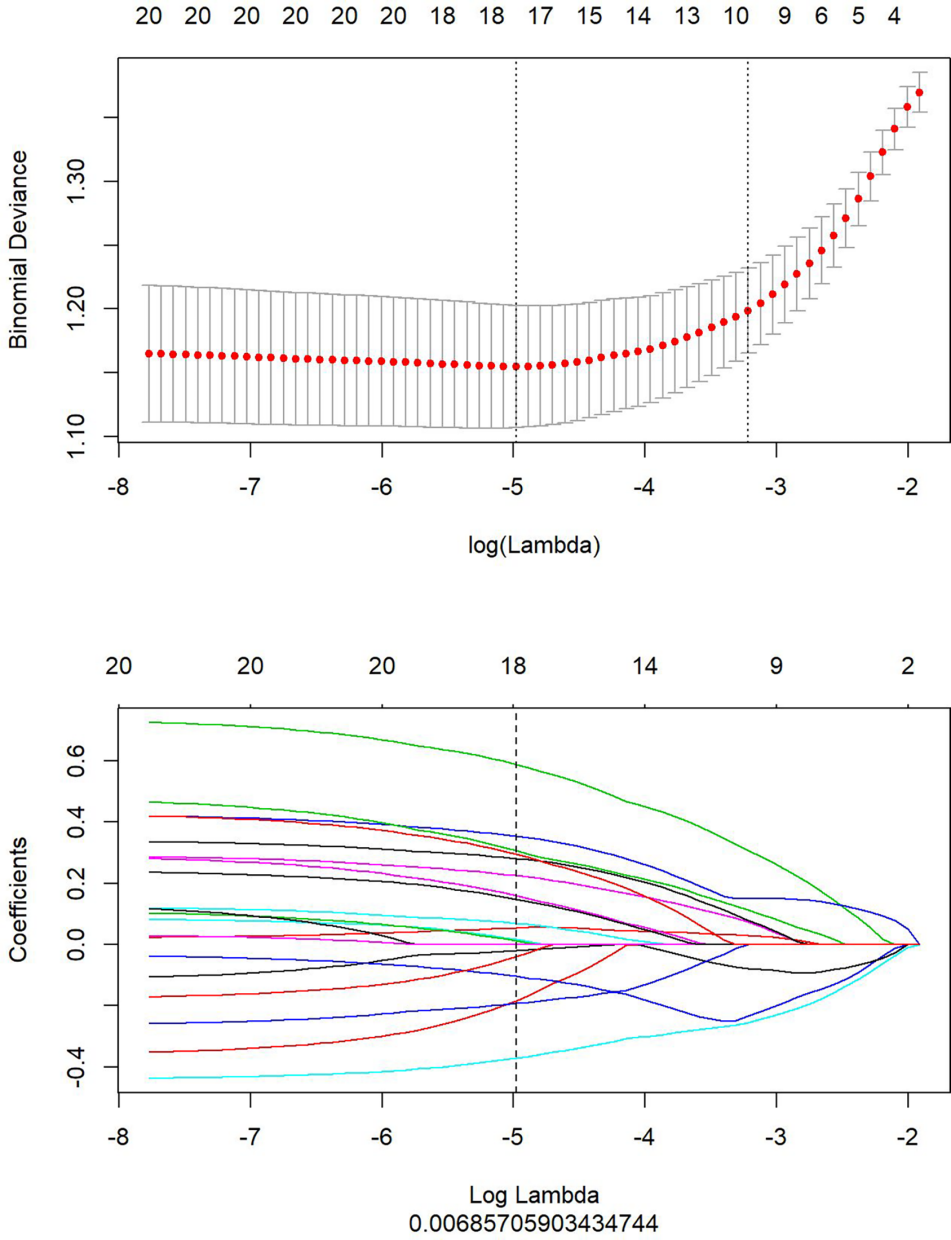
The optimal feature subsets selected by Least Absolute Shrinkage and Selection Operator (LASSO) analysis from the T2-weighted imaging (T2WI) sequence to construct the final model. The upper part shows the optimal λ selected at the lowest point of the curve by LASSO analysis. The lower part displays the penalty graph of the variation coefficients of the 680 imaging features, with the vertical dotted line indicating that a total of 18 of the most significant features are obtained at the optimal λ value when LogLambda is 0.006919.

**Figure 3:**
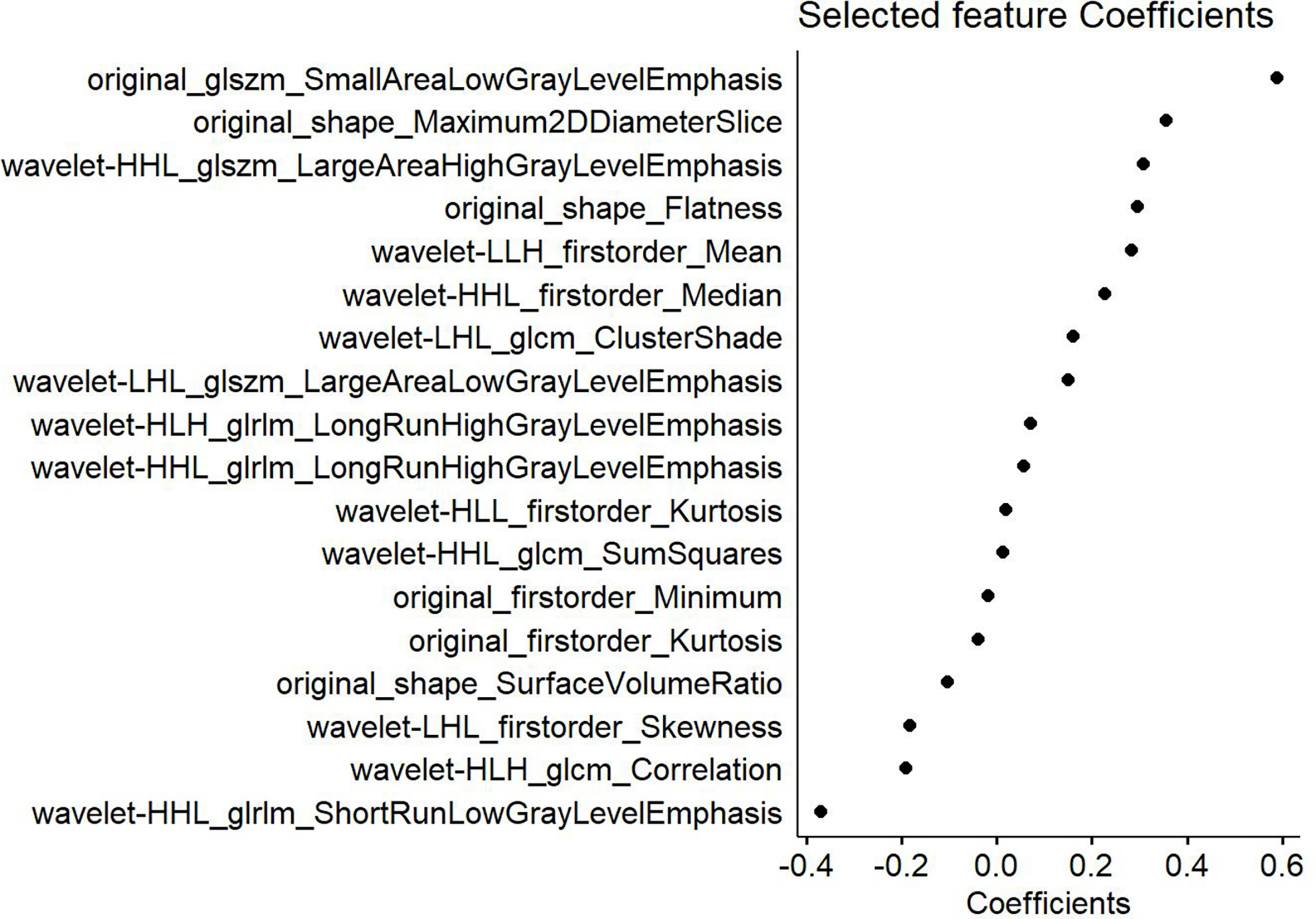
Features selected from the T2-weighted imaging (T2WI) sequence and the corresponding weights. The left part shows the 18 features selected from the T2WI sequence, whereas the right displays the weighting coefficients of the corresponding selected features.

This study extracted 680 imaging features from the T2WI and enhanced T1WI sequences. Subsequently, the redundant and irrelevant features were eliminated using the mRMR algorithm, and the respective top 20 features were retained. Subsequently, LASSO analysis was performed to select the optimal feature subsets used to construct the final model. First, the optimal λ was selected at the lowest point of the curve, and the corresponding λ coefficient was adopted to determine the number of features. After the LASSO regression analysis, the corresponding T2WI λ value of 0.0069 was used to select 18 feature subsets (**Figure 2**). The corresponding coefficients were then evaluated (**Figure 3**) to obtain the Rad-score formula (1).

**Figure 4:**
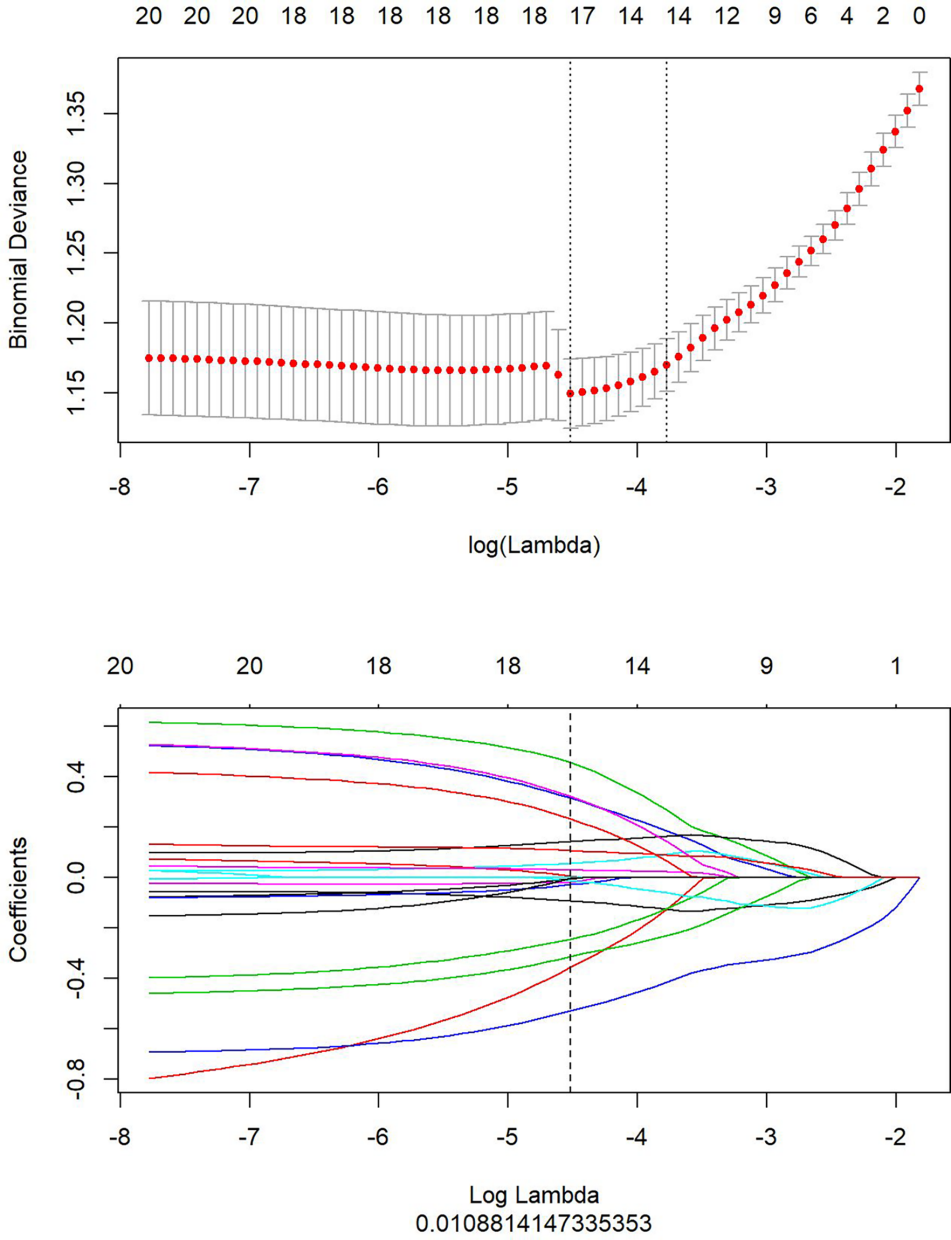
Optimal feature subsets selected from the enhanced T1-weighted imaging (T1WI) sequence using Least Absolute Shrinkage and Selection Operator (LASSO) analysis to construct the final model. The upper part shows the optimal penalty coefficient (λ) selected at the lowest point of the curve by LASSO. The lower part displays the penalty graph of the variation coefficients of the 680 imaging features, with the vertical dotted line indicating that a total of 19 of the most significant features are obtained at the optimal λ value when LogLambda equals 0.01088.

**Figure 5:**
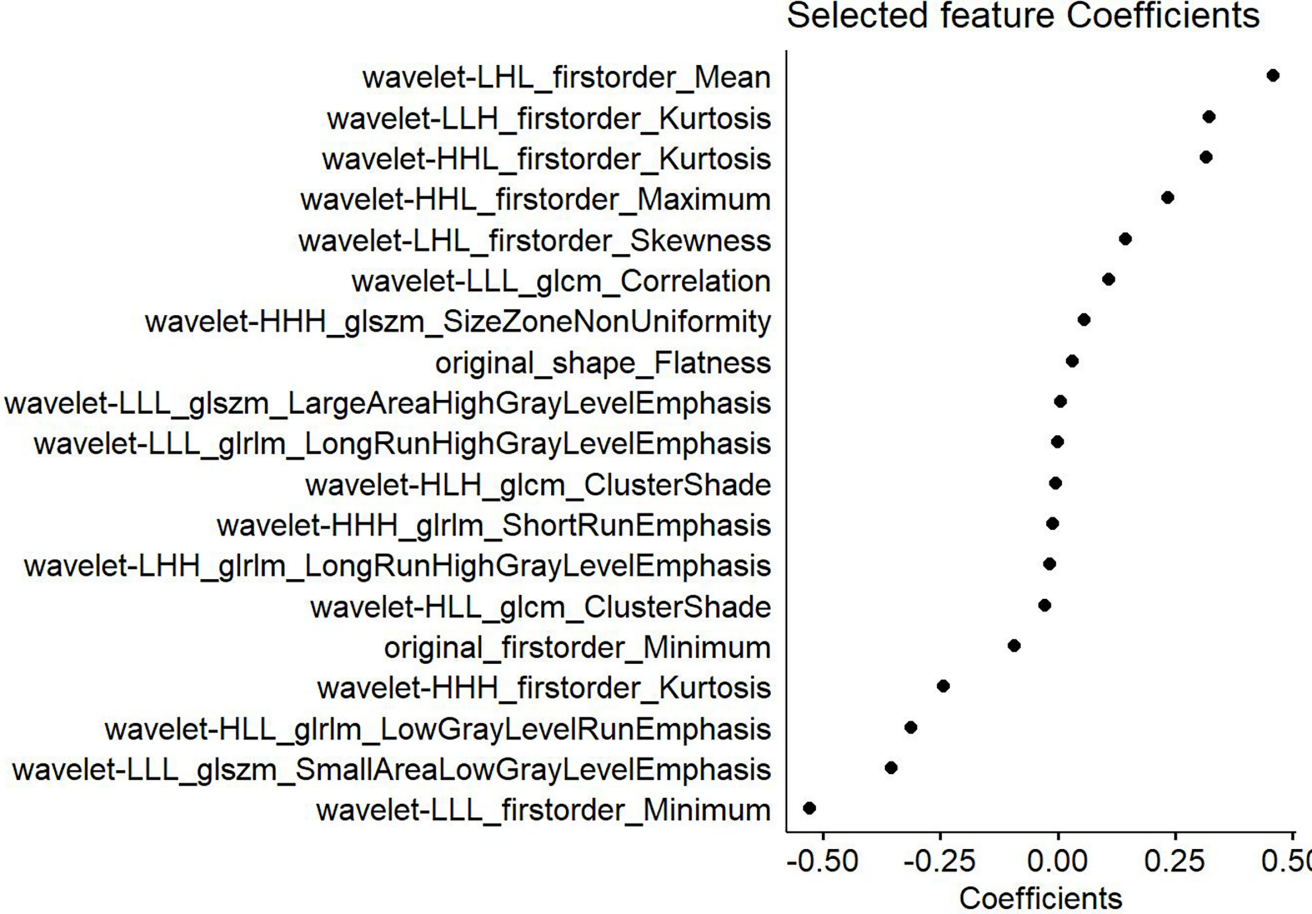
Features selected from the enhanced T1-weighted imaging (T1WI) sequence and the weights of the corresponding features.

Similarly, the optimal λ was selected at the lowest point of the curve in the enhanced T1WI sequence. The corresponding λ of 0.01088 was used to determine the 19 most characteristic therapeutic effect-related subsets after NACT (**Figure 4**). The corresponding subset weights were subsequently evaluated (**Figure 5**), and the Rad-score formula (Equation 3) was obtained.

### Radiomics Model Construction

This study compared the Rad-score values between class 0 (the response group) and class 1 (the non-response group) for the training set and test set based on the T2WI sequence. For the T2WI sequence, there were significant differences in the Rad-score values between the response group and the non-response group in the training set [−0.6 (−1.6, 0.1), 0.2 (−0.4, 0.9), *p* < 0.0001] and test set [−0.8 (−1.7, 0.1), 0.4 (−0.2, 1.3), *p* < 0.0001]. From the T2WI sequence, 18 texture features were selected to differentiate the response group and the non-response group after NACT. In the training set, the AUC value of the radiomics nomogram was 0.76 (95%CI, 0.69–0.83), whereas in the test set, the AUC value was 0.77 (95%CI, 0.67–0.87).

For the enhanced T1WI sequence, this study compared the Rad-score values between the response group and the non-response group for the training set and test set. For the enhanced T1WI sequence, there were significant differences in Rad-scores between the response group and the non-response group in the training set [−0.7 (−1.4, −0.2), 0.2 (−0.3, 0.9), *p* < 0.0001] and test set [−1 (−1.6, −0.4), 0.1 (−0.6, 0.8), *p* < 0.0001]. From the enhanced T1WI sequence, 19 texture features were selected to differentiate the response group from the non-response group after NACT. In the training set, the AUC value of the radiomics nomogram was 0.77 (95%CI, 0.70–0.84), whereas in the test set, the AUC value was 0.75 (95%CI, 0.64–0.86).

### Construction of the Clinical Radiomics Nomograms

**Figure 6:**
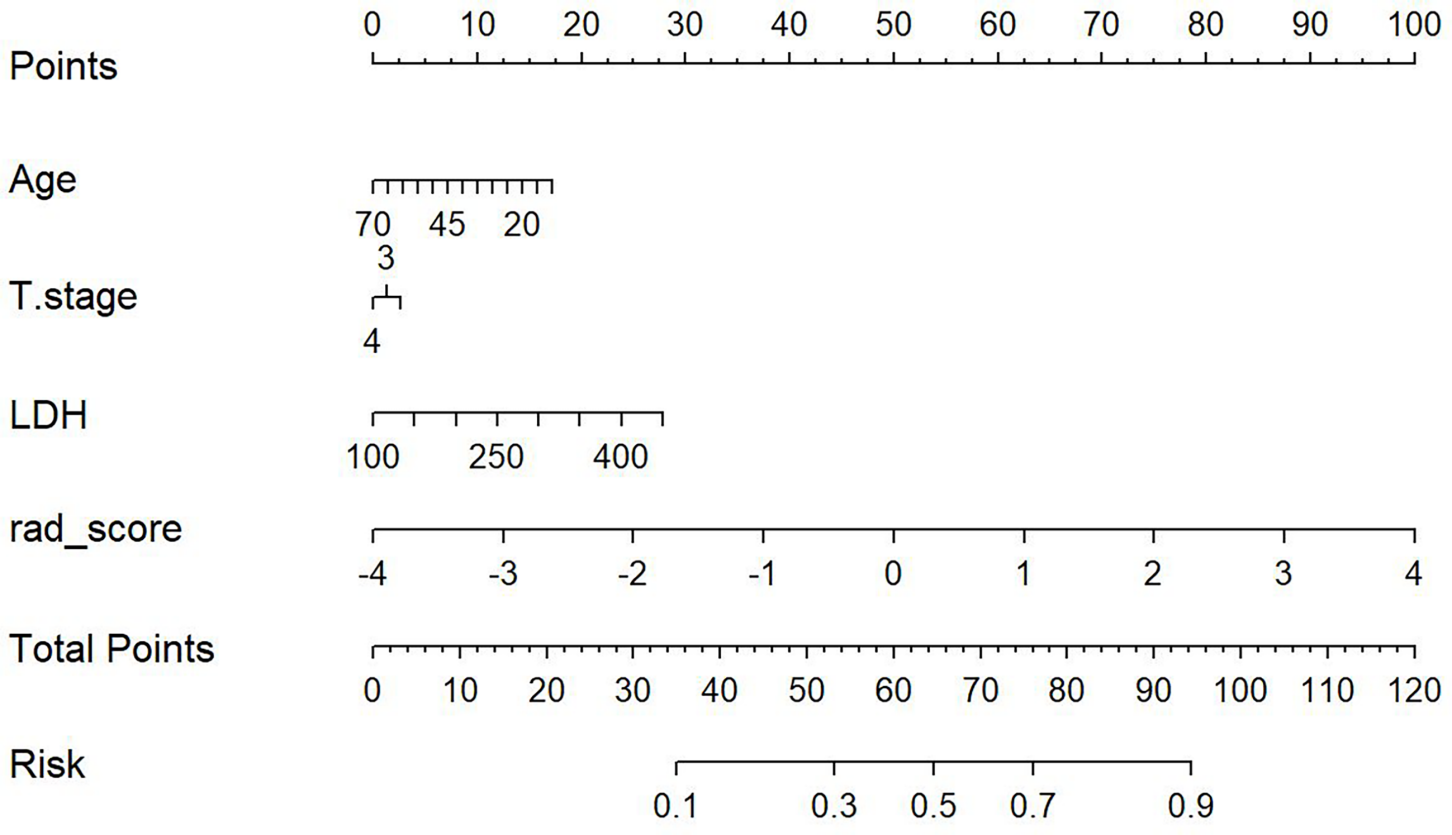
Construction of the model nomogram based on age, tumor (T) stage, and lactate dehydrogenase (LDH) level combined with the T2-weighted imaging (T2WI) radiomics score (Rad-score).

**Figure 7:**
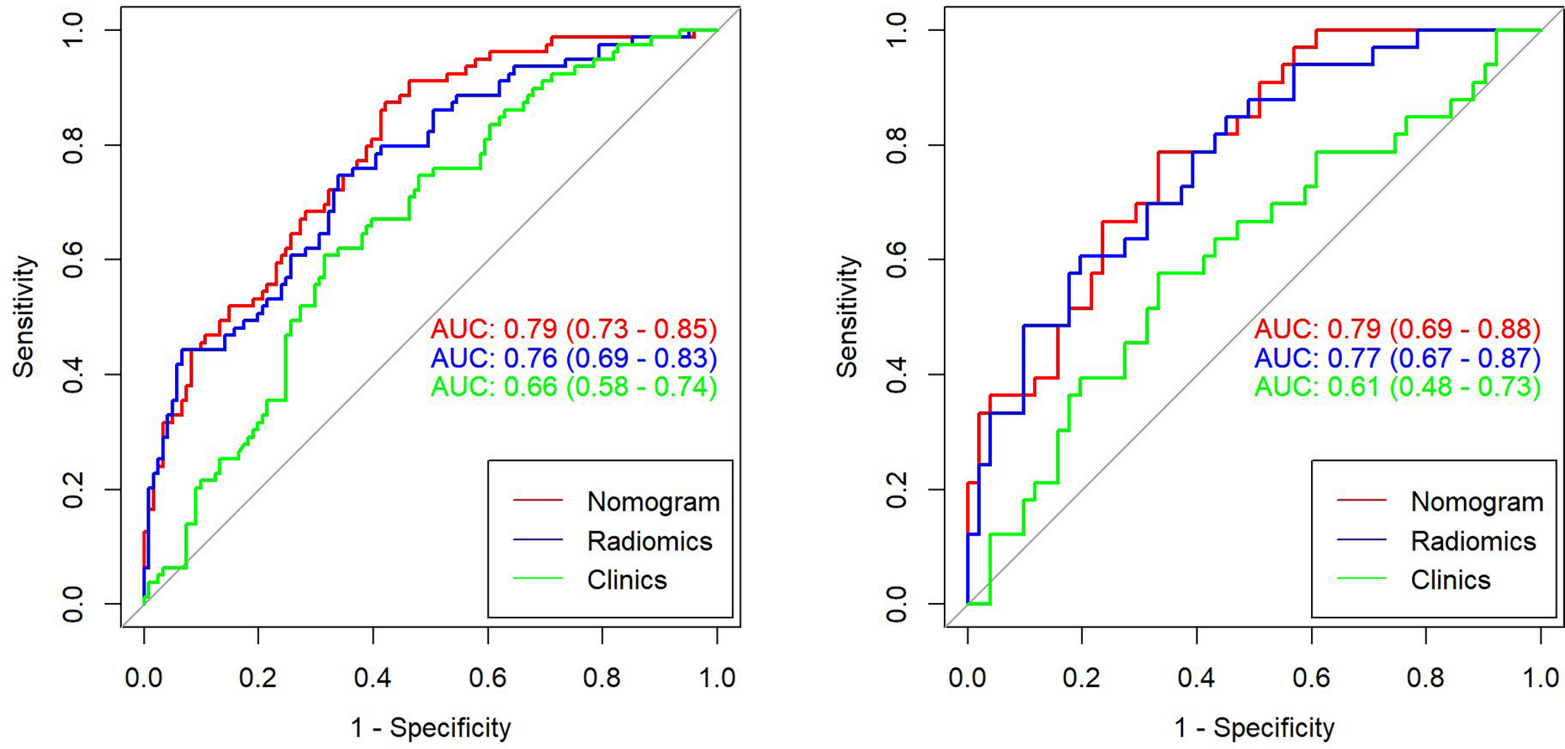
Receiver operating characteristic (ROC) curves for the clinical model, radiomics model, and clinical radiomics nomogram of the training set and test set based on the T2-weighted imaging (T2WI) sequences. The left side shows the training set, whereas the right side shows the test set. The figure indicates that the radiomics and clinical radiomics models had greater area under the curve (AUC) values than the clinical model.

**Figure 8:**
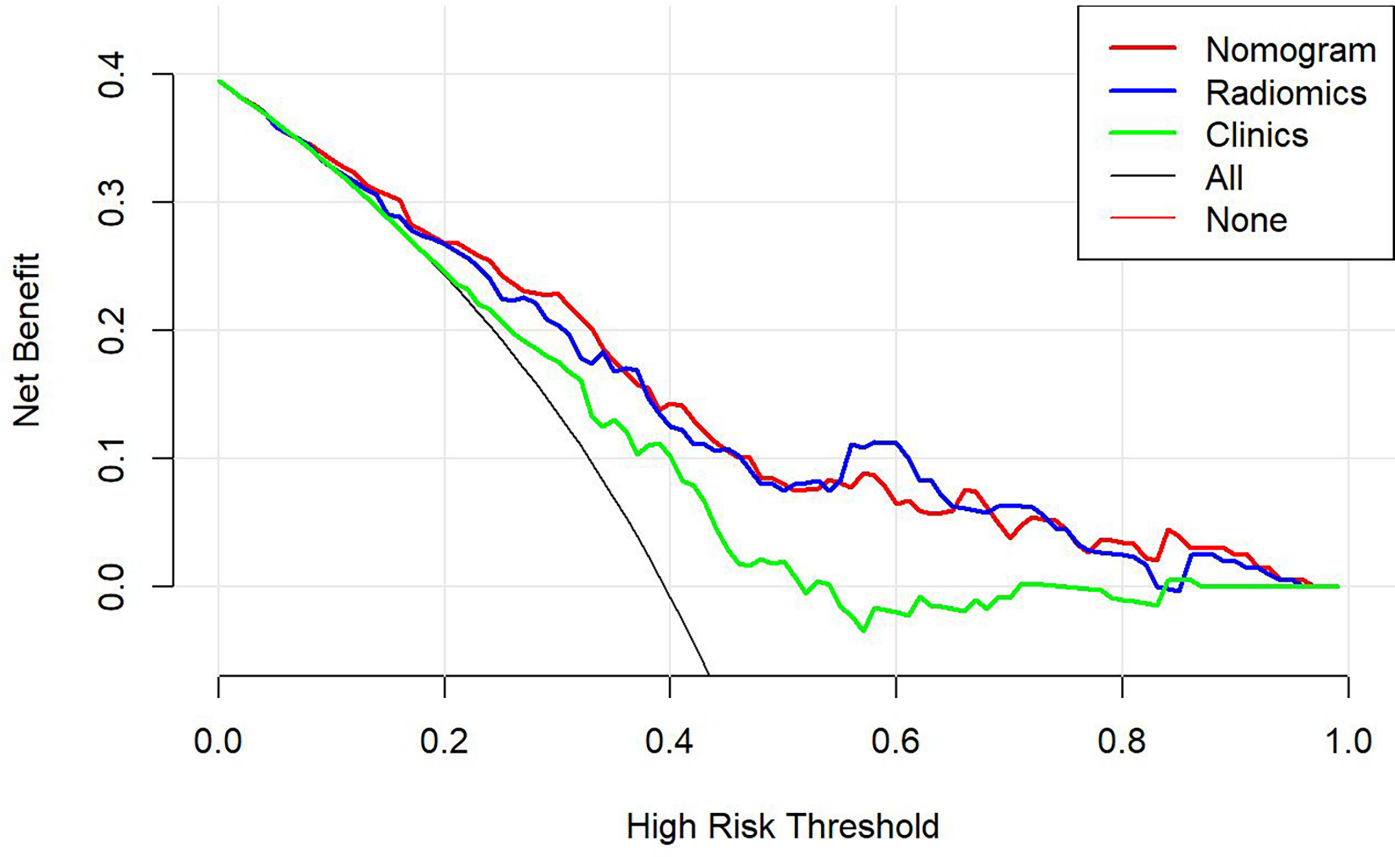
The decision-making curves of the radiomics, clinical radiomics, and clinical models based on the T2-weighted imaging (T2WI) sequence, where the X- and Y-axes represent the threshold probability and net benefit, respectively. Red indicates the clinical radiomics model, blue stands for the radiomics model, and green represents the clinical model. The Y-axis represents the 0-parallel line, indicating that all samples were negative (assuming that all patients were in the non-response group after treatment). The black line is based on the assumption that all samples were in the response group after treatment, and the net benefit is represented by the diagonal line with a negative slope. The net benefit is the proportion calculated by subtracting the false positive count from the true positive count. The threshold probability (Pt) was within the range of 0.1–0.8. The radiomics and clinical radiomics nomograms had a higher Pt and corresponding net benefits than the clinical model, revealing that a higher clinical net benefit was obtained in predicting the therapeutic effect of neoadjuvant chemotherapy.

This study incorporated clinical data (sex, T stage, AJCC stage, EBV-DNA, Rta-IgG, VCA-IgA, EA-IgA, and HGB levels, and PLT counts) in the construction of the nomogram. Based on the radiomics model constructed from the T2WI sequence, patients in the training set and test set were randomly assigned to the constructed clinical nomograms, with age, T stage, and LDH level as the independent risk factors to evaluate the therapeutic effect of NACT. For the clinical nomogram, the AUC value of the training set was 0.66 (95%CI, 0.58–0.74), whereas the AUC of the test set was 0.61 (95%CI, 0.48–0.73). For the T2WI sequence-based radiomics model, the AUC of the training set was 0.76 (95%CI, 0.69–0.83), whereas that of the test set was 0.77 (95%CI, 0.67–0.87). In addition, clinical radiomics nomograms were constructed based on the clinical risk factors and Rad-scores (**Figure 6**), for which the AUC of the training set was 0.79 (95%CI, 0.73–0.85) and the AUC of the test set was 0.79 (95%CI, 0.69–0.88). The Hosmer–Lemeshow test of the clinical radiomics nomograms revealed no significant statistical differences (*p* = 0.8696 for the training set, *p* = 0.6665 for the test set; *p* > 0.05 indicated favorable model fitting). In addition, the DeLong test was used to compare the ROC curves of the clinical radiomics nomogram, the radiomics model, and the clinical nomogram (**Figure 7**). In the training set, the AUC value of the clinical radiomics nomogram was superior to that of the clinical nomogram (*p* = 0.001279); similarly, the AUC value of the radiomics model was greater than that of the clinical model (*p* = 0.03026), although there was no significant difference between the AUCs of the clinical radiomics nomogram model and the radiomics model (*p* = 0.1167). These findings were subsequently verified in the test set. To be specific, the clinical radiomics nomogram had a larger AUC than that of the clinical model (*p* = 0.007617), the radiomics model had a larger AUC value than that of the clinical model (*p* = 0.02188), and there was no significant difference between the AUCs of the clinical radiomics nomogram model and the radiomics model (*p* = 0.6238). The DCA (**Figure 8**) revealed that the radiomics model and clinical radiomics nomogram achieved a higher clinical net benefit in predicting the therapeutic effect of NACT.

**Figure 9:**
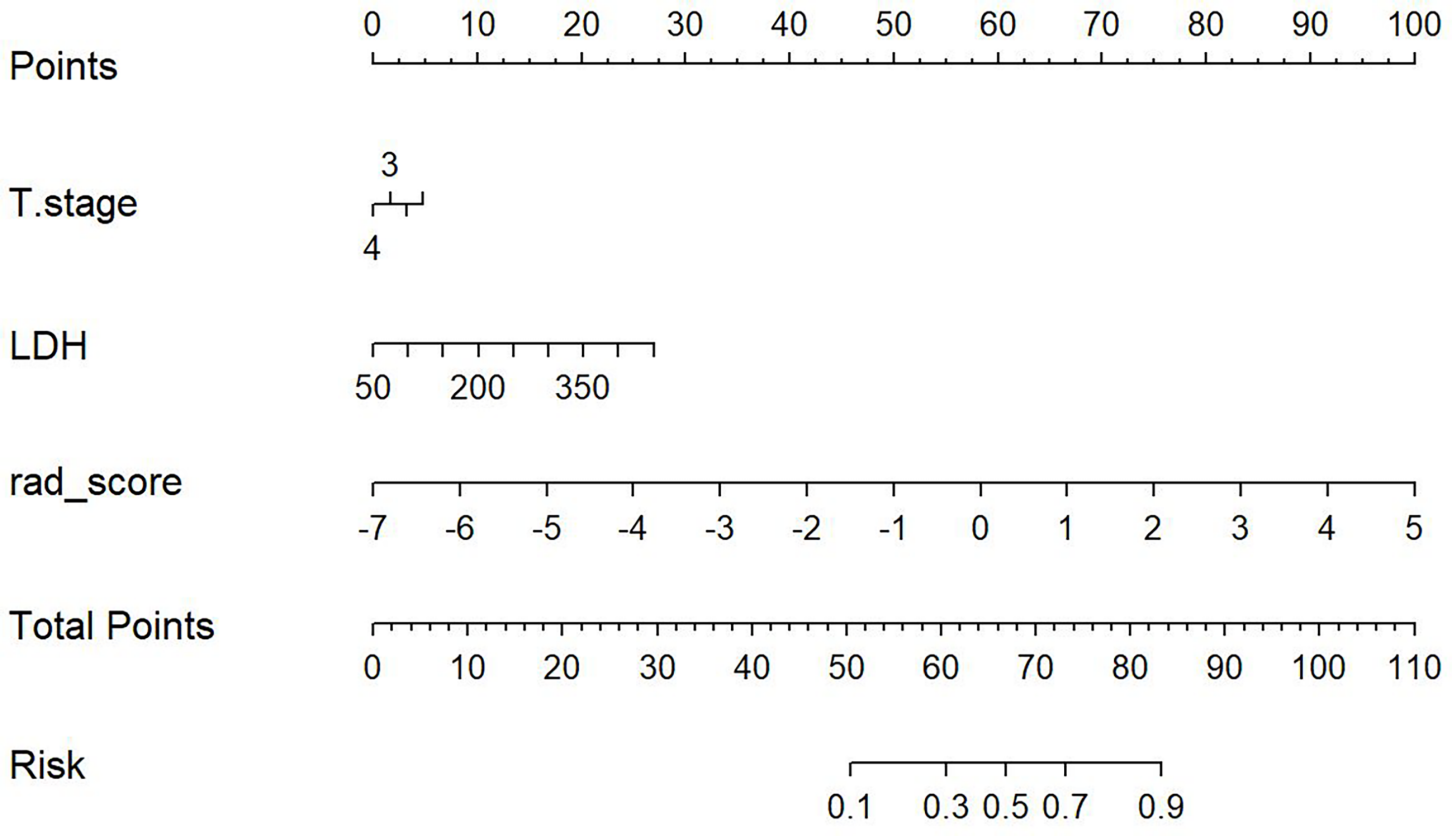
Construction of the model nomogram based on the tumor (T) stage and lactate dehydrogenase (LDH) level in combination with the enhanced T1-weighted imaging (T1WI) sequence.

**Figure 10:**
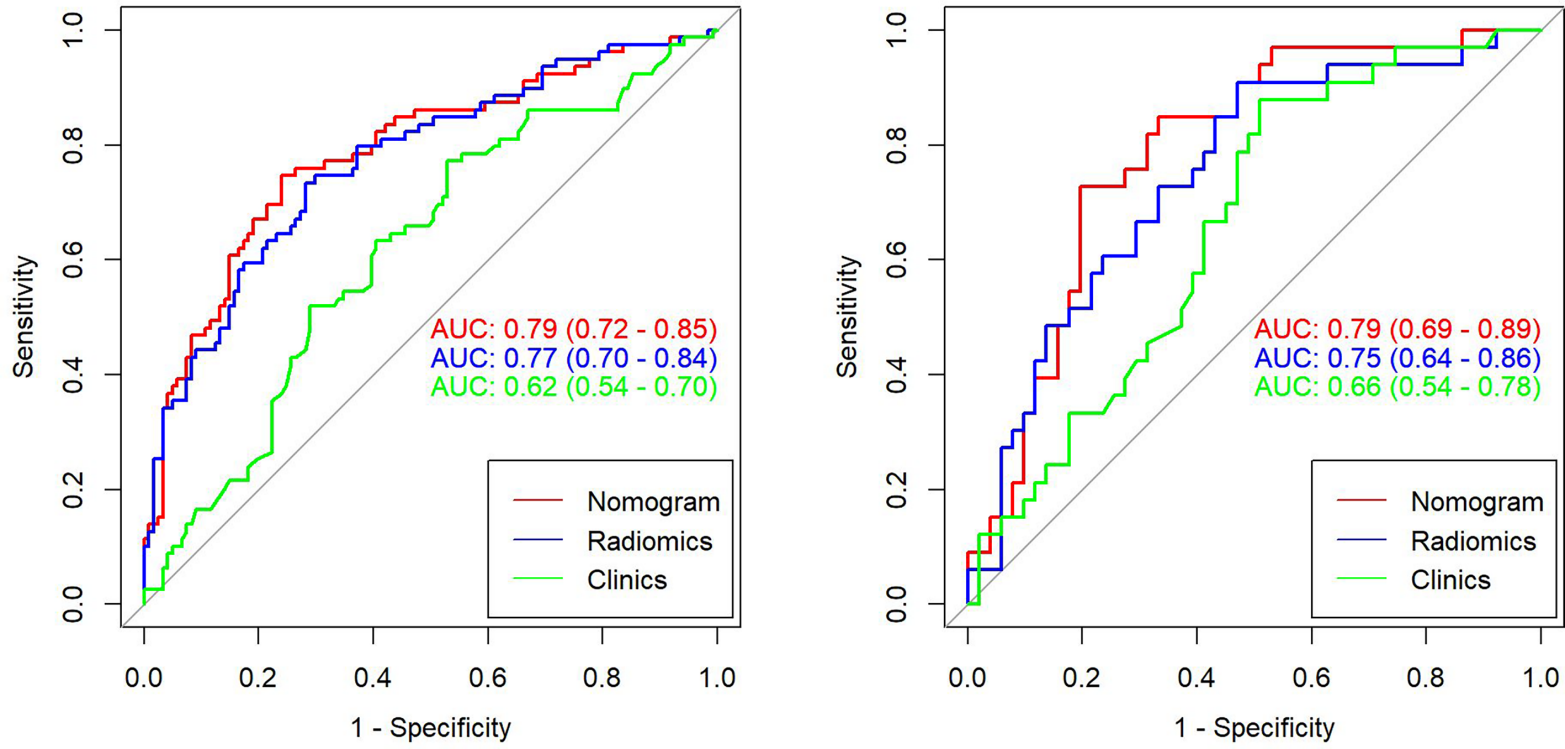
Receiver operating characteristic (ROC) curves for the clinical model, radiomics model, and clinical radiomics nomogram based on the T1-weighted imaging (T1WI) sequence for the training set (left) and test set (right).

**Figure 11:**
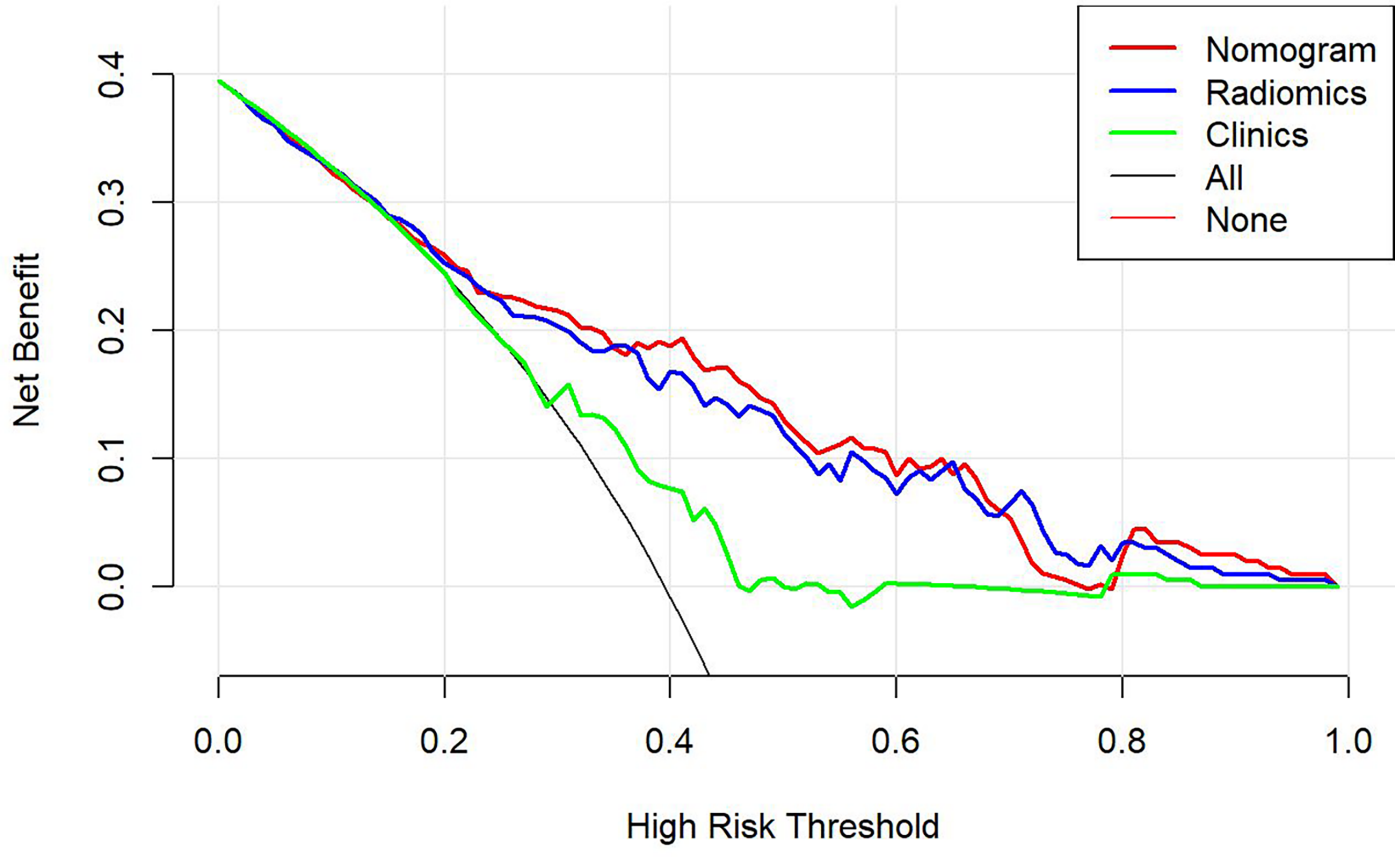
The decision-making curves of the radiomics, clinical radiomics, and clinical models based on the T1-weighted imaging (T1WI) sequence, where the X- and Y-axes represent the threshold probability and net benefit, respectively. Red indicates the clinical radiomics model, blue stands for the radiomics model, and green represents the clinical model. The Y-axis represents the 0-parallel line, indicating that all samples were negative (assuming that all patients were in the non-response group after treatment). The black line is based on the assumption that all samples were positive (in the response group after treatment), and the net benefit is represented by the diagonal line with a negative slope. The net benefit is the proportion calculated by subtracting the false positive count from the true positive count. The threshold probability (Pt) was within the range of 0.2–0.8. The clinical radiomics nomogram had a higher Pt and corresponding net benefits than the clinical model, indicating that it attained a higher clinical net benefit in predicting the therapeutic effect of neoadjuvant chemotherapy.

For the enhanced T1WI sequence, the T stage and LDH level were the independent risk factors for evaluating the therapeutic effect of NACT. For the clinical model, the AUC value of the training set was 0.62 (95%CI, 0.54–0.70), and for the test set, the AUC was 0.66 (95%CI, 0.54–0.78). For the enhanced T1WI sequence, the AUC values of the training set and test sets of the radiomics model were 0.77 (95%CI, 0.70–0.84) and 0.75 (95%CI, 0.64–0.86), respectively. The clinical risk factors and Rad-score were used to construct the clinical radiomics nomogram (**Figure 9**). In the clinical radiomics nomogram, the AUC of the training set was 0.79 (95%CI, 0.72–0.85), and the AUC of the test set was also 0.79 (95%CI, 0.69–0.89). The Hosmer–Lemeshow test revealed no obvious statistical difference in the clinical radiomics nomogram model (*p* = 0.319 for the training set and *p =* 0.9414 for the test set). Upon testing of the goodness of fit of the logistic regression model by the Hosmer–Lemeshow test, the *p*-value was >0.05, indicating good model fitting. The DeLong test or bootstrap testing was used to compare the ROC curves among the three models (**Figure 10**). In the training set, the AUC value of the clinical radiomics nomogram was superior to that of the clinical model (Z = 3.9345, *p* = 0.0000834); similarly, the AUC value of the radiomics nomogram was greater than that of the clinical model (Z = 3.4646, *p* = 0.0005311), and there was no significant difference in the AUC values of the training sets between the clinical radiomics and radiomics models (Z = 1.4504, *p* = 0.1469). In the test set, the clinical radiomics nomogram was superior to the clinical model (Z = 2.1828, *p* = 0.02905), and there was no significant difference between the radiomics and clinical models (Z = 1.3166, *p* = 0.188) or between the clinical radiomics and radiomics models (Z = 1.2304, *p* = 0.2185). The DCA (**Figure 11**) suggested that the clinical radiomics nomogram had a higher clinical net benefit than the clinical model.

## Discussion

Predicting the therapeutic effect of NACT in patients with locally advanced NPC (stages III and IVa) using MR-based radiomics features before treatment has rarely been reported in the literature (26), and predictions based on pre-NACT MR radiomics combined with clinical feature nomograms are even rarer. In the present study, 18 and 19 features related to the chemotherapeutic efficacy were selected from the T2WI and enhanced T1WI sequences, respectively. The results suggested that the clinical radiomics nomogram could function as a means of risk stratification and may be useful for guiding NACT regimens. Moreover, the clinical radiomics nomogram and the T2WI sequence-based radiomics were more likely to predict the prognostic outcomes than the clinical model, suggesting that they may become novel and effective tools to help treat locally advanced NPC in the future.

This study indicated that for the clinical nomogram that was constructed based on the T2WI sequence, the patient’s age, T stage, and LDH level might be the independent risk factors for evaluating the therapeutic effect of NACT. However, the clinical radiomics nomogram and the radiomics model were superior to the clinical model alone. Wang et al. (26) previously investigated T1WI, T2WI, and T2WI fat suppression sequences combined with radiomics, demonstrating an AUC as high as 0.822 (0.809–0.835), which was higher than the AUC obtained from the T2WI sequence or the T2WI sequence-based clinical radiomics nomogram alone in the present study. Some scholars have applied functional magnetic resonance imaging (fMRI) techniques, including DWI, dynamic contrast-enhanced MRI, IVIM, and DKI in their investigations (10–12, 27). Despite the fact that great efforts have been made to predict the therapeutic effect of NACT, most studies are based on the need to frequently measure the tumor at different time points over the course of NACT. In actual practice, it is difficult to frequently examine the lesion, and the measurements cannot accurately reflect the lesion as a whole.

This study revealed that the clinical T stage and LDH level were related to the short-term efficacy of NACT, although the diagnostic predictive values of these measures were low; in fact, both were inferior to the predictive abilities of the T2WI-based clinical radiomics nomogram and the radiomics model. This study constructed the clinical radiomics nomogram prediction model to further investigate the role of T2WI-based radiomics and clinical factors in predicting short-term treatment efficacy. These models may facilitate the more precise delineation of target volumes, aid in conducting radiation dosimetric research, help in informing concurrent radiochemotherapy regimens, and provide prognostic indexes for clinicians to aid in individualized treatments. In this study, 19 features were selected based on the T2WI sequence that had the greatest weights in predicting the efficacy of NACT. Among these features, the small area low gray-level emphasis (SALGLE) of the GLSZM in the original image had the greatest weight, followed by the maximum 2D diameter slice, while flatness ranked fourth in the weighting. After wavelet transformation, the features with the greatest weights were the large area high gray-level emphasis (LAHGLE) in the GLSZM, the mean in the first-order features, the cluster shadow in the GLCM, and the long-run high gray-level emphasis (LRHGLE). The SALGLE feature of the GLSZM measures the distributed proportion of the regional integration with low gray-level values in the image, whereas the LAHGLE feature measures that of the regional integration with high gray-level values in the image. The GLSZM has better performance in describing the texture consistency, for example, mottled or aperiodic textures. However, in terms of image texture, the GLCM outperforms the GLRLM. In this study, the SALGLE and LAHGLE values were both greater in the non-response group than in the response group. When describing lesion brightness and complexity, greater values are indicative of higher NPC lesion heterogeneity and poorer NACT efficacy. The maximum 2D diameter slice feature defines the maximum pairwise Euclidean distance between the tumor surface grid vertices on the transverse plane. According to the results of the present study, the maximum 2D diameter Slice (namely, the maximum tumor diameter of the maximum sectional area) was greater in the non-response group than in the response group. RECIST represents the most important radial line that is generally used to evaluate the efficacy of antitumor therapies (27), which was greater in the non-response group than in the response group. Flatness is a feature that indicates the relationship between the maximum and minimum features within the boundaries of the ROI, with values ranging from 1 (non-planar, spherical) to 0 (planar). In this study, the flatness value in the non-response group was greater than that in the response group, suggesting that the morphology in the non-response group was irregular or more spherical, whereas the value in the response group was closer to that representing a more planar surface. Most features were transformed from wavelets, and the mean in the first-order features describes the average gray-level intensity in the ROI, with greater brightness corresponding to a greater mean. In this study, the mean in the first-order features was greater in the non-response group than in the response group. The cluster shadow in the GLCM is a measure of the skewness and homogeneity of the GLCM, with a higher cluster shadow value indicating a greater asymmetry of the mean. In the present study, the cluster shadow value was greater in the nonresponse group than in the response group, as was the LRHGLE of the GLRLM. The LRHGLE is a measure of the joint distribution of a long run of high gray-level values, with a higher LRHGLE value indicating a coarser texture. The present findings were similar to those of a study conducted by Wang et al. (28), which showed that the LRHGLE value was the most important feature for predicting the recurrence of anal cancer.

In the clinical nomogram that was constructed based on the enhanced T1WI sequence, the T stage and LDH level might serve as the independent risk factors for predicting the efficacy of NACT. The AUC values of the training set and test set of the clinical radiomics nomogram were greater than those of the clinical nomogram model. This study focused on evaluating parameters like the flatness of the original images, the mean/kurtosis/maximum/skewness in the first-order features after wavelet transformation, the relevance of the GLCM, the size-zone non-uniformity (SZN) and LAHGLE in the GLSZM, and the LRHGLE and short run emphasis (SRE) in the GLRLM, all of which had greater values in the non-response group than in the response group. Flatness is related to the tumor mass morphology, the value of which was greater in the non-response group than in the response group. As for the mean/kurtosis/maximum/skewness in the first-order features, the higher peak and more concentrated brightness indicated a greater mean. More obvious increases in these two features in the enhanced T1WI sequence are indicative of more compact tumor cells. Skewness is a measure of the mean asymmetry based on deviations in the distribution of bright areas in the ROI. If the bright region is greater than the gray region in an ROI, the skewness value is positive. In this study, the skewness value was greater in the non-response group than in the response group. The relevance of the GLCM is measured by values ranging from 0 (irrelevance) to 1 (complete relevance) and is a measure used to indicate the degree of linear correlation between the gray value and the individual voxels in the GLCM. The value for this parameter in the non-response group was closer to 1, indicating a more linear correlation, whereas the value in the response group was closer to 0, indicating a higher degree of irrelevance. The SZN in the GLSZM measures the volume variability of the ROI in an image, with a lower value indicating more volume homogeneity in terms of the size of the region. In this study, the SZN value in the non-response group was greater than that in the response group, suggesting a greater degree of heterogeneity in the volume of the region. The LAHGLE parameter is related to the signal intensity of the tumor, with more obvious enhancement and a greater LAHGLE value being indicative of a denser tumor mass. The SRE in the GLRLM measures the short run distribution, with a greater value indicating a shorter run and finer texture, whereas a greater LRHGLE value suggests a coarser texture; both of these values were greater in the non-response group than in the response group.

There were more male than female patients in the present cohort, and the DCA revealed that the clinical radiomics nomogram of each sequence obtained a higher net benefit than the clinical model. In diverse clinical models, the patient’s age, T stage, and LDH level might serve as the independent predictors of the therapeutic effect of NACT. This study found that a more obvious regression could be obtained after NACT in cases of T3 tumors than in T4 tumors. Besides, T4 stage tumors have a larger volume and are more susceptible to liquid necrosis, and chemotherapeutic agents may reach the necrotic area in lower concentrations. Some reports have suggested that the serum LDH level is an independent prognostic factor in the treatment of non-distant metastasis (29). In the present study, the LDH level was also one of the effective clinical predictors of NACT efficacy. Some research indicates that measuring pretreatment plasma EBV-DNA levels can improve the ability to predict the prognosis or response to treatment (30, 31); however, this factor failed to distinguish the response group from the non-response group in the present study. This inconsistency might be ascribed to the inconsistent EBV-DNA detection procedures and the large degree of variability in EBV-DNA levels. Evaluating the T stage, assessing serum LDH levels, and employing MR-based multiparameter radiomics before initiating NACT are factors that might predict the short-term efficacy of NACT, possibly improving clinical outcomes.

Some limitations of this study should be noted. First of all, the data in this study were obtained from patients who were scanned using the same machine at the same center; therefore, external verification using datasets collected from multiple centers is required. Second, this study was retrospective in nature, and after applying the exclusion criteria, all the patients were those with locally advanced NPC (stage III–IVa) who received NACT, which may have resulted in selection bias. Thus, prospective trials with patients with newly diagnosed NPC should be conducted in the future. Third, all of the images used in the study were manually segmented. Due to the extensive NPC invasion range and the complicated skull base structure, it is impossible to automatically or semiautomatically segment the images, resulting in an inevitable degree of subjectivity and poor repeatability of the image segmentation process. Fourth, this study only verified the diagnosis of the primary NPC lesion, and the regional lymph nodes were not evaluated. For this reason, prospective studies should be carried out in the future to analyze changes in the clinical index before and after treatment to optimize the clinical radiomics indexes. Fifth, this study complied with the RECIST standard in the formulation of the therapeutic effect standard. In the future, a more accurate gold standard will be required for further investigation. Finally, this study only investigated changes from a pathophysiological and clinical perspective. Future proteomic and genomic studies are needed to further explore the interactions between the variables evaluated in this study.

## Conclusion

In conclusion, this study constructed and validated an MR radiomics nomogram based on multiple pretreatment clinical indexes. The nomogram may serve as a precise medical index that clinicians can use to predict the efficacy of treatment before initiating NACT in patients with NPC.

## Data Availability Statement

The raw data supporting the conclusions of this article will be made available by the authors, without undue reservation.

## Author Contributions

CH and YC contributed to the conception and design of the study. DZ, XC, YF, and TL collected experimental data, CH and YC created clinical indices of the dataset. CH delineated gross tumor volume for each patient. PP contributed to the computation of image features. CH and PP performed statistical analysis. CH wrote the draft of the manuscript. All authors contributed to the article and approved the submitted version.

## Funding

Contract grant sponsor: Youth Scientific Research Project Foundation of Fujian Province; contract grant number: 2015-1-12; Contract grant sponsor: Fujian Health Commission.

## Conflict of Interest

PP is employed by GE Healthcare.

The remaining authors declare that the research was conducted in the absence of any commercial or financial relationships that could be construed as a potential conflict.

## Publisher’s Note

All claims expressed in this article are solely those of the authors and do not necessarily represent those of their affiliated organizations, or those of the publisher, the editors and the reviewers. Any product that may be evaluated in this article, or claim that may be made by its manufacturer, is not guaranteed or endorsed by the publisher.
